# HBV Pre-S1-Derived Myristoylated Peptide (Myr47): Identification of the Inhibitory Activity on the Cellular Uptake of Lipid Nanoparticles

**DOI:** 10.3390/v13050929

**Published:** 2021-05-17

**Authors:** Masaya Nanahara, Ya-Ting Chang, Masaharu Somiya, Shun’ichi Kuroda

**Affiliations:** 1 The Institute of Scientific and Industrial Research (ISIR-Sanken), Osaka University, Osaka 567-0047, Japan; nanahara44@sanken.osaka-u.ac.jp (M.N.); blair8510@gmail.com (Y.-T.C.); msomiya44@sanken.osaka-u.ac.jp (M.S.); 2 Graduated School of Frontier Biosciences, Osaka University, Osaka 565-0871, Japan; 3 Advanced Photonics and Biosensing Open Innovation Laboratory (PhotoBIO-OIL), AIST-Osaka University, Osaka 565-0871, Japan; 4 College of Life Science, National Taiwan University, Taipei 10617, Taiwan

**Keywords:** hepatitis B virus (HBV), Myr47 lipopeptide, cellular uptake, liposomes, sodium taurocholate cotransporting polypeptide (NTCP), HBV surface antigen (HBsAg), apolipoprotein E (ApoE)

## Abstract

The Myr47 lipopeptide, consisting of hepatitis B virus (HBV) pre-S1 domain (myristoylated 2–48 peptide), is an effective commercialized anti-HBV drug that prevents the interaction of HBV with sodium taurocholate cotransporting polypeptide (NTCP) on human hepatocytes, an activity which requires both N-myristoylation residue and specific amino acid sequences. We recently reported that Myr47 reduces the cellular uptake of HBV surface antigen (HBsAg, subviral particle of HBV) in the absence of NTCP expression. In this study, we analyzed how Myr47 reduces the cellular uptake of lipid nanoparticles (including liposomes (LPs) and HBsAg) without NTCP expression. By using Myr47 mutants lacking the HBV infection inhibitory activity, they could reduce the cellular uptake of LPs in an N-myristoylation-dependent manner and an amino acid sequence-independent manner, not only in human liver-derived cells but also in human non-liver-derived cells. Moreover, Myr47 and its mutants could reduce the interaction of LPs with apolipoprotein E3 (ApoE3) in an N-myristoylation-dependent manner regardless of their amino acid sequences. From these results, lipopeptides are generally anchored by inserting their myristoyl residue into the lipid bilayer and can inhibit the interaction of LPs/HBsAg with apolipoprotein, thereby reducing the cellular uptake of LPs/HBsAg. Similarly, Myr47 would interact with HBV, inhibiting the uptake of HBV into human hepatic cells, while the inhibitory effect of Myr47 may be secondary to its ability to protect against HBV infection.

## 1. Introduction

Hepatitis B virus (HBV) is a DNA virus specifically infecting the human liver hepatocyte, and causes serious liver diseases such as hepatitis, cirrhosis, and liver cancer. Although HBV was discovered nearly half a century ago, the curative agent has not yet been developed [1], and only HB vaccine can prevent the horizontal and vertical transmission of HBV. HBV is an enveloped virus containing three types of envelope proteins called S, M, and L protein that share the same C-terminal sequence [2]. Notably, the pre-S1 region of the L protein is essential for specific recognition of human hepatocytes [3,4,5]. N-terminal 3–77 amino acid residues (aa) of the pre-S1 region [6] and myristoyl residue at N-terminus of the pre-S1 region [7,8] are indispensable for the cell entry process. Recently, an essential region for hepatocyte recognition was identified as 9–18 aa of the pre-S1 region [9]. In 2012, the sodium taurocholate cotransporting polypeptide (NTCP) that is specifically expressed in hepatocyte was identified as a critical cellular receptor for HBV early infection (cell entry step) [10].

As a promising agent for the prevention of HBV infection, a pre-S1-derived peptide encompassing 2–48 aa with N-myristoyl residue (hereafter Myr47) can inhibit the cellular entry of HBV by repressing the interaction between NTCP and HBV in a competitive manner [11]. Currently, Myr47 and its derivatives (Hepcludex, Bulevirtide, and Myrcludex-B) are on the market in EU countries for the treatment of chronic HDV patients with HBV superinfection.

Our group reported that Myr47 inhibits the cellular uptake of HBsAg (HBV surface antigen, subviral particle of HBV) by human hepatocellular carcinoma-derived Hep G2 cells (no NTCP expression) [11]. In this study, we examined if Myr47 inhibits the cellular uptake of lipid nanoparticles (liposomes (LPs) and HBsAg) by Hep G2 cells in the absence of NTCP. We investigated the effect of N-myristoyl residue and the amino acid sequence of Myr47 on the inhibition of cellular uptake. In addition, since ApoE3 (human apolipoprotein E3) was shown to play an important role in the cellular uptake of HBV [12] and the elimination of LPs from blood by hepatocytes [13], we found that the N-myristoyl residue of Myr47 inhibits the formation of the LPs-ApoE3 complex.

## 2. Materials and Methods

### 2.1. Materials

Biotinylated Myr47 lipopeptide was synthesized by Scrum. Biotinylated mutated Myr47 lipopeptides (aa2–48, D-11,13, Ala11–15, Scrambled (Scr) [14]) were purchased from Biologica, Nagoya, Japan. DOPC (1,2-Dioleoyl-sn-glycero-3-phosphocholine) was purchased from NOF, Tokyo, Japan. Cholesterol and CellVue Claret were purchased from Sigma-Aldrich, St. Louis, MO, USA. DiD (1,1′-dioctadecyl-3,3,3′,3′-tetramethylindodicarbocyanine, 4-chlorobenzenesulfonate salt) was purchased from Thermo Fisher Scientific, Waltham, MA, USA. Anti-FITC IgG and anti-human apolipoprotein E3 IgG were purchased from GeneTex, Irvine, CA, USA. 18:1 FITC cap PE (1,2-dioleoyl-sn-glycero-3-phosphoethanolamine-N-(carboxyfluorescein) (ammonium salt)) was purchased from Avanti Polar Lipids, Alabaster, AL, USA. AlphaScreen protein A detection kit was purchased from PerkinElmer, Waltham, MA, USA. Human apolipoprotein E3 (ApoE3) was purchased from Peprotech, Rocky Hill, NJ, USA. Hepatitis B patients’ plasma-derived HBsAg particles and mouse monoclonal anti-S IgG (clone824) were purchased from the Institute of Immunology.

### 2.2. Cell Culture

Human hepatocellular carcinoma Hep G2 cells, HuH-7 cells, human embryonic kidney HEK293 cells, and human cervical adenocarcinoma HeLa cells were obtained from JCRB Cell bank and cultured in DMEM (Dulbecco’s modified Eagle medium) containing 10% FBS (fetal bovine serum), 100 U/mL penicillin, and 100 μg/mL streptomycin at 37 °C in 5% CO_2_ humidified incubator.

### 2.3. Liposomes (LPs)

Each of DOPC, cholesterol, DiD, and 18:1 FITC cap PE was dissolved in ethanol as 1 mg/mL. Lipids solutions were mixed (DOPC: cholesterol = 6:4 (mol/mol) with/without 0.1 mol% of DiD or 2 mol% of 18:1 FITC cap PE) and injected into the 9-fold volume of PBS (phosphate-buffered saline) with vortexing. Subsequently, the mixture was sonicated by a Branson sonifier 450 at RT (room temperature) for 20 s and used immediately. The diameter and zeta potentials of LPs were measured by dynamic light scattering method using a Nanosizer (Malvern, Malvern, UK). LPs used in this study are approximately 61 nm in diameter, PDI (particle diversity index) = 0.195, and −0.649 mV zeta potentials.

### 2.4. Confocal Laser Microscopy

Hep G2 cells (2 × 10^5^ cells/well) were inoculated in an 8-well glass bottom chamber one day before the experiment. Immediately after Myr47 (500 nM) injection, DiD-labeled LPs (10 μg/mL) were added to the wells. After 3 h of incubation, the cells were washed twice with PBS, and fixed by 4% (*w*/*v*) paraformaldehyde in PBS (400 μL/well) containing Hoechst 33342 (2 μg/mL) for 20 min. The cells were subsequently washed once with PBS, and the Fluoro-KEEPER Antifade Reagent (nacalai tesque) was added to the wells. The cells were observed under confocal laser microscopy FV1000 (Olympus, Tokyo, Japan).

### 2.5. Flow Cytometry

Hep G2 cells (1.5 × 10^5^ cells/well) were inoculated in a 12-well plate one day before the experiment. The medium was changed to DMEM without FBS. Immediately after ApoE3 (25 μg/mL) injection, Myr47 (500 nM) was added to the medium, and then DiD-labeled LPs (5 μg/mL) or CellVue-labeled HBsAg (5 μg/mL as protein) was added to the medium. After 3 h incubation, the medium was removed, and the cells were detached with 5 mM EDTA in PBS and subjected to Flow cytometer FACS Canto II (BD Bioscience, San Jose, CA, USA). Data were analyzed by the FlowJo7.6.5 program. For labeling HBsAg with CellVue Claret, HBsAg (500 μg/mL as protein) in PBS was incubated with the same volume of CellVue solution (250-fold dilution) at RT for 5 min and mixed with 4-fold volume of DMEM containing 10% FBS to stop labeling reaction.

### 2.6. Alphascreen^®^ Assay

An AlphaScreen protein A detection kit was used with Streptavidin beads. A mixture of 1 M NaCl, 1 M Tris-HCl (pH = 8), and 1% (*w*/*v*) BSA (bovine serum albumin) was used as the sample buffer. FITC-LPs (300 ng) and either Myr47 or its mutants (50 ng) in 15 μL of the sample buffer were incubated in a 384-well microplate at RT for 30 min. Subsequently, for fixation of LPs on protein A beads, anti-FITC IgG (2 ng) in 5 μL sample buffer was injected into each well. Then, the plate was incubated at RT for 30 min. After that, protein A beads and Streptavidin beads (0.1 μL each) in 10 μL of the sample buffer were added to each well, and the plate was incubated at RT for 1 h. The interaction between the LPs and Myr47 or its mutants was quantified by measuring the fluorescence intensity. As for the interaction between HBsAg and Myr47 or its mutants, HBsAg (50 ng as protein) and anti-S domain IgG (2 ng) were used instead of FITC-LPs and anti-FITC IgG, respectively.

### 2.7. Bio-Layer-Interferometry (BLI) Analysis

Unless otherwise described, all operations were performed with BLItz (Fortebio, Fremont, CA, USA) at RT with agitation at 2200 rpm. Anti-Human ApoE3 IgG (1 μM) was contacted with a protein A sensor chip (Fortebio) for 240 s. The sensor chip was washed with K-buffer (PBS containing 0.1% BSA) for 30 s. ApoE3 (2.5 μM) could bind with the antibody on the sensor chip for 120 s. The sensor chip was washed with K-buffer for 120 s. Meanwhile, mixture of LPs (500 μg/mL) and Myr47 or its mutants (50 μg/mL) was incubated at RT for 20 min and put in 4-μL well. The fabricated sensor chip was immersed in the sample solution, and the frequency change (nm, corresponding to binding) was measured for 360 s.

## 3. Results

### 3.1. Inhibition of Cellular Uptake of Liposomes (LPs) by Myr47

Since the cellular uptake of HBsAg by Hep G2 cells (no NTCP expression) was efficiently inhibited by Myr47 [11], we speculated that this inhibitory activity was not directly related to HBV envelope proteins, but to the lipid nanoparticle structure. In this study, we therefore tried to use LPs, instead of HBsAg and HBV as a starting point for the subsequent analyses.

**Figure 1:**
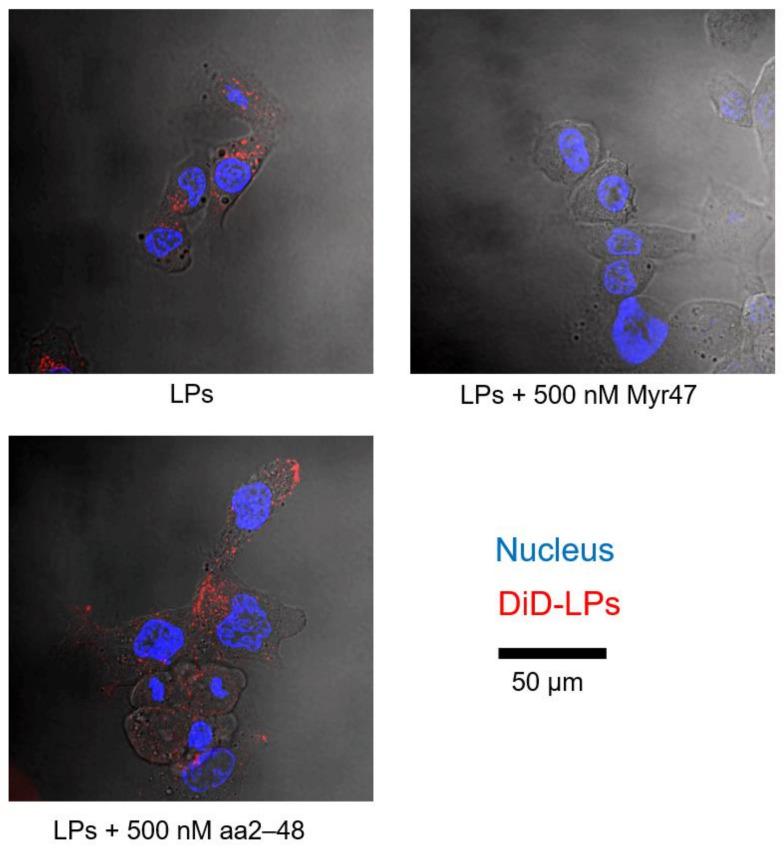
Inhibitory effect of Myr47 on the cellular LP uptake by Hep G2 cells (no NTCP expression). Hep G2 cells were incubated with DiD-LPs (10 μg/mL) in serum-free DMEM containing Myr47 or its mutant (500 nM) for 3 h at 37 °C, and then observed under confocal laser scanning microscopy. Nucleus (cyan) were stained with Hoechst 33342 (2 μg/mL) and LPs (red) were stained with DiD.

**Figure 2:**
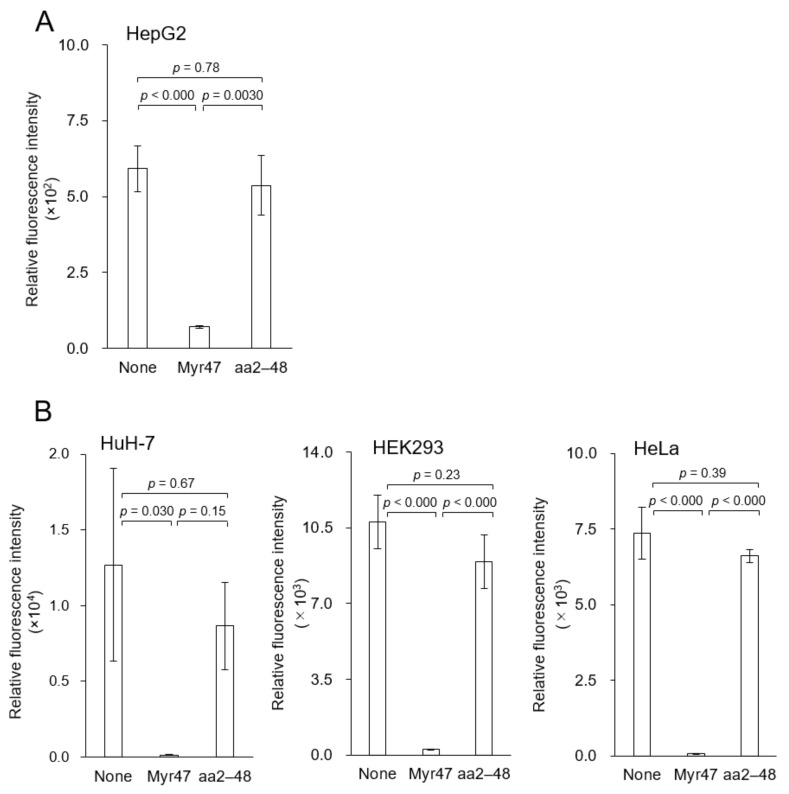
Inhibitory effect of Myr47 on the cellular LP uptake by various cells (no NTCP expression). (**A**) Hep G2 cells. (**B**) Huh-7 cells, HEK293 cells, and HeLa cells. Cells were incubated with DiD-LPs (5 μg/mL) in serum-free DMEM containing Myr47 or its mutant aa2–48 (500 nM) for 3 h at 37 °C, and then analyzed with flow cytometry. Data are shown as means ± S.D. (*n* = 3).

When DiD (fluorophore)-labeled LPs were incubated with Hep G2 cells (no NTCP expression) for 3 h, efficient cellular uptake of LPs was observed under confocal laser microscopy (Figure 1, left upper panel). The addition of 500 nM of Myr47 was found to inhibit the cellular uptake of LPs (right upper panel). Flow cytometric analysis also demonstrated that 500 nM of Myr47 inhibited nearly 90% of the cellular uptake of LPs (Figure 2A). Together, as previously observed with HBsAg [11], Myr47 can inhibit the cellular uptake of LPs independent of NTCP expression.

Next, we evaluated if N-myristoylation affects the inhibition activity of Myr47 by using N-myristoyl residue-less Myr47 mutant, aa2–48, which cannot inhibit the cellular entry of HBV [14]. While Myr47 inhibited the uptake of LPs, aa2–48 could not inhibit the uptake (Figure 1, left lower panel; Figure 2A). This result suggested that N-myristoyl residue is indispensable for the inhibition of cellular uptake of LPs. Furthermore, similar LP uptake inhibitory activity was observed in various human cells (no NTCP expression), such as other human liver-derived cells (Huh-7 cells) and human non-liver cells (HEK293 cells and HeLa cells) (Figure 2B). The biological activity of these lipopeptides was found to be functional independent of cell type and NTCP expression.

### 3.2. N-Myristoylation-Dependent Interaction between Myr47 and LPs

**Figure 3:**
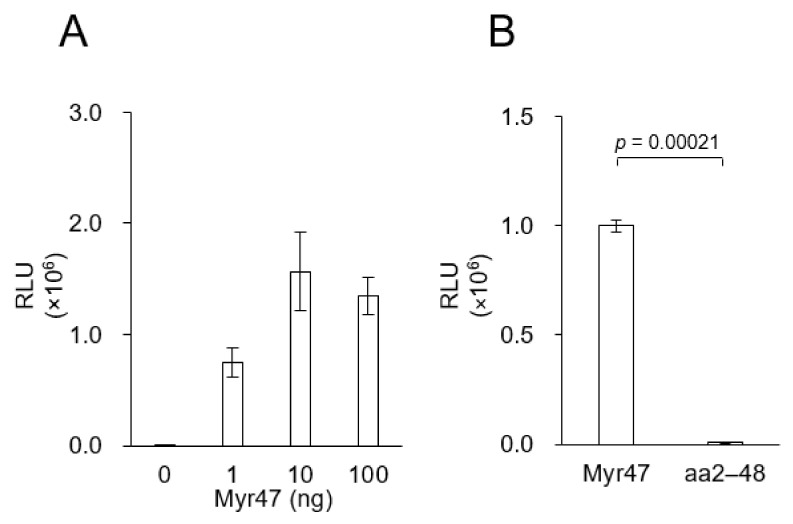
In vitro direct interaction of Myr47 with LPs. (**A**) Interaction of Myr47 with LPs analyzed by AlphaScreen assay. (**B**) Interaction of Myr47 or aa2–48 with LPs analyzed by AlphaScreen assay. Data are shown as means ± S.D. (*n* = 3).

Based on the above results, we considered that Myr47 interacts with LPs and thereby interferes the cellular uptake of LPs in an NTCP-independent manner; therefore, we examined the interaction of Myr47 with LPs by using an AlphaScreen based on Amplified Luminescent Proximity Homogeneous Assay [15]. Both FITC-labeled LPs and biotin-labeled Myr47 were fixed on anti-FITC IgG-conjugated protein A acceptor beads and streptavidin donor beads, respectively. As shown in Figure 3, the interaction of Myr47 with LPs gave strong luminescence, which was a comparable level of positive control, while Myr47 mutant aa2–48 gave no luminescence. These results strongly suggested that Myr47 can anchor to the lipid bilayer of LPs with myristoyl residue like other N-myristoylated peptides [16].

### 3.3. Effect of Amino Acid Sequence on the Myr47-Mediated Inhibitory Activity

**Figure 4:**
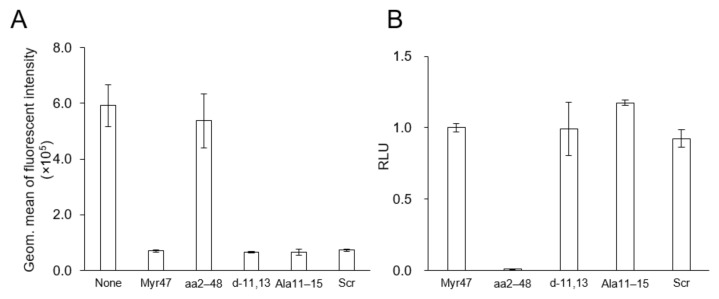
Effect of Myr47 and its mutants on the cellular LP uptake activity and the in vitro direct interaction with LPs. (**A**) Hep G2 cells were incubated with DiD-LPs (5 μg/mL) in serum-free DMEM containing Myr47 or its mutants (500 nM) for 3 h at 37 °C and then analyzed with flow cytometry. (**B**) Interaction of Myr47 or its mutants with LPs analyzed by AlphaScreen assay. Data are shown as means ± S.D. (*n* = 3).

Various Myr47 mutants have been examined to inhibit the establishment of HBV infection (mainly, cellular entry of HBV) [14]. In this study, three N-myristoylated Myr47 mutants that lost HBV infection inhibitory activity (i.e., Ala11–15, d-11,13, Scrambled) and one N-myristoyl residue-less Myr47 (i.e., aa2–48) were synthesized (Table 1). Under the same condition of Figure 2, these mutants were examined for the cellular uptake of LPs by using flow cytometric analysis (Figure 4A). All N-myristoylated Myr47 mutants inhibited the cellular uptake of LPs, whereas the N-myristoyl residue-less Myr47 mutant could not inhibit the cellular uptake. We then analyzed the interaction between these Myr47 mutants and LPs by using AlphaScreen as described above (Figure 3). All N-myristoylated Myr47 mutants could interact with LPs efficiently to similar extents of Myr47 (Figure 4B). These results indicated that the inhibitory activity of Myr47-mediated cellular LP uptake requires N-myristoyl residue but does not require the Myr47 specific sequence and the NTCP expression.

### 3.4. Effect of Myr47 on the ApoE3 (Apolipoprotein E3)-LPs Interaction

**Figure 5:**
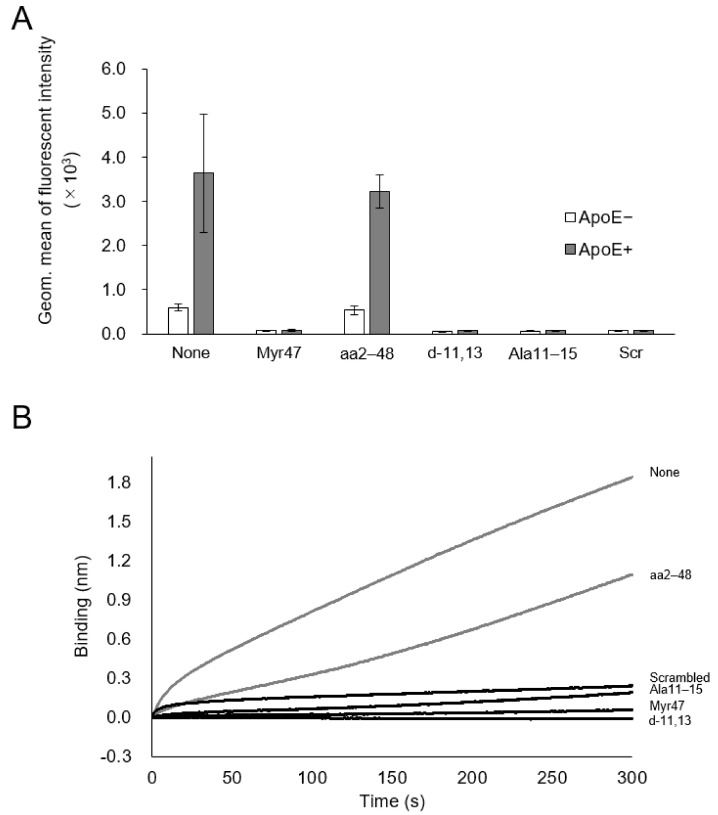
Effect of Myr47 and its mutants on the ApoE3-LP interaction. (**A**) Effect of ApoE3 on the cellular LP uptake activity in the presence of Myr47 or its mutants. Hep G2 cells were incubated with DiD-LPs (5 μg/mL) in serum-free DMEM containing Myr47 or its mutants (500 nM) with/without ApoE3 (25 μg/mL) for 3 h at 37 °C, and then analyzed with flow cytometry. Data are shown as geometric means ± S.D. (*n* = 3). (**B**) Effect of Myr47 or its mutants on the interaction of LPs and ApoE3 was evaluated with BLItz. Data are shown as means. (*n* = 3).

Recently, ApoE3 was reported as an indispensable factor to establish HBV infection, presumably enhancing the cellular entry of HBV by forming ApoE3-HBV complex [12]. To decipher the mechanism underlying Myr47-mediated inhibition of cellular LP uptake, Hep G2 cells (no NTCP expression) were incubated in serum-free medium (not containing ApoE) and contacted with DiD-labeled LPs for 3 h in the presence of Myr47 or its mutants with/without 25 μg/mL ApoE3. Cellular uptake of LPs was measured by flow cytometric analysis (Figure 5A). When ApoE3 was added to the medium, the cellular uptake of LPs was increased up to 6-fold compared with the cellular uptake obtained without the addition of ApoE3. The addition of Myr47 or N-myristoylated mutants completely inhibited the uptake of LPs, while N-myristoyl residue-less mutant (aa2–48) showed no inhibition.

Next, we measured the in vitro direct interaction between LPs and ApoE3 using the bio-layer interferometry (BLI) method. ApoE3 was fixed on the protein A sensor chip bridged by anti-ApoE3 IgG. The sensor chip was dipped into LP solution containing Myr47 or its mutants. Myr47 and its mutants, except aa2–48, apparently inhibited the interaction between LPs and ApoE3, whereas aa2–48 inhibited to some extent (Figure 5B). These results corroborated that ApoE3 enhances the cellular uptake of LPs by forming an ApoE3-LP complex. N-myristoylated peptide, especially N-myristoyl residue, could interfere with the formation of ApoE3-LP complex, thereby inhibiting the cellular uptake of LPs.

### 3.5. Effect of Myr47 on the Cellular Uptake of HBsAg

**Figure 6:**
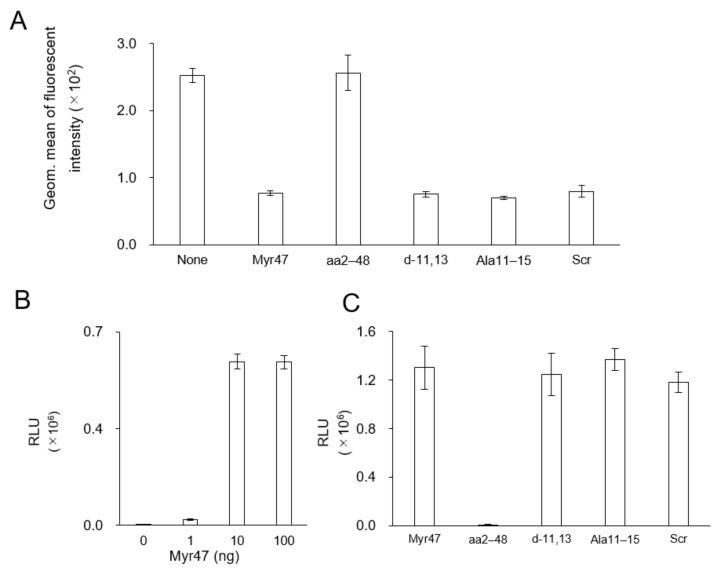
Effect of Myr47 and its mutants on the cellular HBsAg uptake activity and the in vitro direct interaction with HBsAg. (**A**) Hep G2 cells were incubated with CellVue-labeled HBsAg (5 μg/mL as protein) in serum-free DMEM containing Myr47 or its mutants (500 nM) for 3 h at 37 °C, and then analyzed with flow cytometry. (**B**) Interaction of Myr47 with HBsAg analyzed by AlphaScreen assay. (**C**) Interaction of Myr47 or its mutants with HBsAg analyzed by AlphaScreen assay. Data are shown as means. (*n* = 3).

Myr47 was shown to inhibit the cellular uptake of HBsAg (HBV subviral particle) by Hep G2 cells (no NTCP expression) [11]. Herein, we examined if Myr47 mutants affect the cellular uptake of HBsAg by flow cytometric analysis under the same condition (see Figure 4A). Myr47 mutants (except aa2–48) could inhibit almost 70% of cellular HBsAg uptake, which is a comparable level of My47 (Figure 6A). Next, the interaction of Myr47 or its mutants with HBsAg was measured by AlphaScreen under the same condition (see Figure 3 and Figure 4B). Both HBsAg and Myr47 were fixed onto anti-S (main constituent of HBsAg) IgG-conjugated protein A acceptor beads and streptavidin donor beads, respectively. Myr47 was found to interact with HBsAg efficiently (Figure 6B). Myr47 mutants (except aa2–48) also interacted with HBsAg in an N-myristoyl residue-dependent manner (Figure 6C), as well as the LP-Myr47 interaction, suggesting that N-myristoyl residue anchors to the lipid bilayer of HBsAg.

We attempted to analyze the ApoE3-HBsAg interaction by BLI method but it was not successful. Because the HBsAg used was derived from HB patients’ plasma, it is thought that blood borne ApoE binds to HBsAg, and that this ApoE interferes with the BLI assay. As a corroboration, the cellular uptake inhibition of HBsAg by Myr47 (approximately 70% inhibition, see Figure 6A) was lower than that of LPs (approximately 90% inhibition, see Figure 4A).

## 4. Discussion

Our group recently reported that Myr47 inhibits the uptake of HBsAg by Hep G2 cells (no NTCP expression) [11]. In this study, flow cytometric analysis revealed that the cellular LP uptake by Hep G2 cells was inhibited by Myr47 and its mutants. The inhibitory activity depends on the N-myristoyl residue but not on the amino acid sequence of lipopeptides. This inhibitory activity was inferred to be general for lipopeptides, independent of cell type and NTCP expression. Subsequent analysis by AlphaScreen revealed that Myr47 and its mutants interact with LPs in an N-myristoyl residue-dependent manner. Meanwhile, ApoE3 enhanced the uptake of LPs by Hep G2 cells, which was strongly inhibited by N-myristoyl residue of Myr47 and its mutants. Furthermore, the interaction between ApoE3 and LPs was inhibited by Myr47 and its mutants in an N-myristoyl residue-dependent manner but not in an amino acid sequence-dependent manner.

ApoE3 can interact with the surface of lipid nanoparticles (i.e., LPs, remnant lipoproteins) through a lipid-binding site residing on the C-terminal half and promote the endocytosis of lipid nanoparticles via heparan sulfate proteoglycan (HSPG), low-density lipoprotein receptor (LDLr), or LDLr-related protein (LRP) [13,17]. Since N-myristoylated peptides are spontaneously anchored to lipid bilayers of LPs [18], we herein hypothesized that Myr47 and its mutants anchored on the surface of lipid nanoparticles increased steric hindrance and then caused the inhibition of ApoE3 binding to the surface of nanoparticles. To our knowledge, for the first time it is indicated that the fatty acyl chain of lipopeptides contributes to the inhibition of apolipoprotein-mediated endocytosis by modifying the surface of lipid nanoparticles. When therapeutic agent-containing lipid nanoparticles are used as a DDS (drug delivery system) nanocarrier targeting specific organs/cells in vivo, the surface of nanocarriers are usually modified only with targeting molecules (e.g., antibodies, homing peptides, sugar chains). These DDS nanocarriers are unexpectedly trapped by scavenger receptors (i.e., LDLr, LRP) in the reticuloendothelial system. Dual surface modification of DDS nanocarriers with targeting molecules and lipopeptides may improve the delivering efficiency to target organs/cells in vivo.

We examined the effects of Myr47 and its mutants on the cellular uptake of HBV subviral particle (HBsAg; as a mimic of HBV) by Hep G2 cells (no NTCP expression). These lipopeptides interacted with the HBsAg surface through their myristoyl residue and inhibited the cellular uptake of HBsAg in an N-myristoyl residue-dependent manner, not in an amino acid sequence-dependent manner. HBV (HBsAg as well) was shown to interact with ApoE3 [12]. Hence, we speculated that lipopeptides inhibit the uptake of HBsAg by repelling the access of ApoE3 to HBsAg; likewise, in the case of LPs. However, unlike LPs, the addition of ApoE3 did not enhance the uptake of HBsAg by Hep G2 cells (data not shown). Since the HBsAg used is derived from patients’ plasma and probably formed a complex to some extent with bloodborne ApoE3, the cellular uptake of HBsAg would not be sufficiently enhanced.

In the early stages of HBV infection, HBV initially interacts with cell-surface HSPG, enters the endosomes, interacts with NTCP, sheds its envelope, and releases its components into the cytoplasm for establishing HBV infection. It has been widely accepted that Myr47 prevents the establishment of HBV infection by inhibiting the interaction of HBV with NTCP in a competitive manner [19], and the inhibitory activity of Myr47 depends on both amino acid sequence and myristoyl residue. In this study, Myr47 and its mutants could inhibit the cellular uptake of HBsAg by Hep G2 (no NTCP expression) in a myristoyl residue-dependent manner and not in an amino-acid sequence-dependent manner. Since Myr47 effectively inhibits the establishment of HBV infection, whereas its mutants do not [14], the former activity of Myr47 plays a pivotal role in the prevention of HBV infection, whereas the latter activity certainly contributes to the prevention of HBV infection but is considered to be less important.

Recently, we found that in at least seven cell types (including human liver-derived Huh-7 cell) liposomes are endocytosed, resulting in the formation of numerous lipid droplets [20]. We also identified scavenger receptor class B type 1 (SR-B1) as the receptor for liposome uptake in HEK293 cells [21,22]. Furthermore, the phenomenon of lipid droplet formation in HepG2 cells stimulated with fatty acids has been reported as an in vitro model of steatosis formation [23]. In summary, lipopeptides containing Myr47 may be useful for steatosis prevention. On the other hand, the risk of developing nonalcoholic fatty liver disease (NAFLD) in HBV-infected patients has been found to be significantly lower than in healthy individuals [24], but how Myr47, preventing HBV infection, affects NAFLD prevention and reversal should await further studies.

## Figures and Tables

**Table 1:** Myr47 and its mutant peptides.

Name	Sequence
Myr47	Myr-GTNLSVPNPLGFFPDHQLDPAFGANSNNPDWDFNPNKDQWPEANQVK-biotin
aa2–48	GTNLSVPNPLGFFPDHQLDPAFGANSNNPDWDFNPNKDQWPEANQVK-biotin
Ala11–15	Myr-GTNLSVPNPAAAAADHQLDPAFGANSNNPDWDFNPNKDQWPEANQVK-biotin
d-11,13	Myr-GTNLSVPN$\underset{*}{P}$L$\underset{*}{G}$FFPDHQLDPAFGANSNNPDWDFNPNKDQWPEANQVK-biotin
Scrambled (Scr)	Myr-TNNDPFKRTDLAWNGFDSVAQNDLLPNPPFNNWGDGSHQPADAKPFTK-biotin

Mutated residues were underlined. *D*-amino acids were marked with asterisks. The amino acid residue composition of Myr47 and Scrambled (Scr) lipopeptide is almost identical.
